# Substitute running outputs in elite youth male soccer players: less peak but greater relative running outputs

**DOI:** 10.5114/biolsport.2023.112969

**Published:** 2022-03-16

**Authors:** Michael G. Sydney, Martin Wollin, Dale Chapman, Nick Ball, Jocelyn K. Mara

**Affiliations:** 1 University of Canberra Research Institute for Sport and Exercise (UCRISE), Canberra, Australia; 2 School of Science, Faculty of Science & Technology, University of Canberra, Australia; 3 Discipline of Sport and Exercise Science, Faculty of Health, University of Canberra, Australia; 4 School of Allied Health, Curtin University, Perth, Australia; 5 Performance Health Management, Canberra, Australia; 6 School of Health Sciences, La Trobe University, Melbourne, Australia

**Keywords:** Association Football, High-Speed Running, GPS, Team Sports, Player Monitoring, Physical Performance

## Abstract

Coaches consider substitute players to be a substantial factor in influencing the outcome of a soccer match. Substitute players are expected to make physical impact on the match by superseding the running output of the player they replaced and are a key tool for managing in-game fatigue and influencing the outcome of a game. This study investigated the physical impact and internal response of substitute players, compared to starting and full-match players. We also sought to determine if differences between substitution statuses were influenced by playing position. Players wore 15-Hz global positioning system tracking devices across 29 competition matches and were categorised according to their substitution status (full-match, starters, substitutes) and playing position (external defender, midfield, external attacker and central attacker). Peak total (TD) and high-speed running (> 5.0 m/s) distance (HSRD) were calculated using 1-, 2- and 5-minute rolling epochs. Relative running demands were reported as TD and HSRD per minute of total playing time. Substitute players performed less peak TD and HSRD in 1-, 2- and 5-minute epochs, and reported lower RPE compared to starting and full-match players. In contrast, substitutes performed greater relative HSRD per minute than starting and full-match players (p < 0.001, |d| range = 0.35–1.34). In conclusion, substitute players may have a relative physical impact but do not replicate or supersede the peak demands of full-match players. Coaches and practitioners should implement targeted warm-up interventions to enhance substitute readiness to meet the peak running demands in order to have a more effective physical impact.

## INTRODUCTION

During regulation soccer matches, coaches from each team are permitted to replace up to three on-field players from a group of 6–7 reserve players. Once a player has been substituted, they may not return to the field of play. Prior studies have demonstrated that players’ total and high-speed running distance per minute progressively declined over the course of match-play due to fatigue [1–4]. The tactical use of substitutions is a key mechanism for coaches to counteract the effects of game-induced fatigue to avoid decreases in individual and team performance [3–5]. The physical impact of substitutes can be deemed effective when the substitute players physical work rate supersedes the player being replaced [4–6]. There is often an assumption by coaches that substitutes have a positive physical impact on a match [5], however, studies detailing the running demands of elite youth soccer players commonly omit match contributions by substitutes [2, 7–10], and therefore the empirical evidence for this assumption is limited.

Research detailing match contributions from full-match, starting and substitute players in elite senior male soccer demonstrated that substitutes performed more relative total distance per minute and high-speed running (5.0–6.95 m/s) distance per minute compared to players who started or completed the match [3, 11]. However, previous investigations have also highlighted key differences in the running demands between elite senior and youth male soccer players [12]. For example, elite youth male soccer players have been found to cover greater total distances than elite senior players during match-play, although this was primarily attributed to larger distances covered at low-intensity (< 3.33 m/s) [12]. Alternatively, elite senior male soccer players have demonstrated more distance covered at high-speed (> 6.94 m/s) compared to their youth counterparts, likely attributed to their greater physical capabilities [12]. The results from these studies suggest that data pertaining to the influences of contextual factors such as substitution status in elite senior male soccer players cannot be inferred and used to understand the physical demands of match-play in elite youth male soccer players.

The physical demands of soccer match-play are typically reported as absolute (e.g., total distance covered in a match) and relative (e.g., average distance covered per minute of match time) values to highlight both the total external load and average intensity of players [9, 10, 12]. However, several investigations have demonstrated that utilising absolute and relative metrics may underestimate the most intense periods of match play faced by players [8, 13–15]. As such, the use of methodologies to identify the peak running demands of match-play (such as fixed [e.g., 0–5 min, 5–10 min, 10–15 min] and rolling [e.g., 0–5 min, 1–6 min, 2–7 min] epochs [i.e., time intervals]) have become increasingly common, with the intent to better understand the physical requirements of players [8, 13–15].

Practically, provided that players are adequately prepared for pitch entry, coaches should be able to make tactical player replacements with the aim that substitute players are able to attain similar or greater running outputs as starting and full-match players [5, 16–18]. The use of Global Positioning Systems (GPS) has facilitated the quantification of peak and relative running demands and further aided in the understanding of the external loads of match-play [9, 13–15]. However, given that substitute players experience reduced match-time compared to those who complete an entire match or are in the starting line-up, it is also important to assess how players respond to such external loads [19–21]. Measurements such as rate of perceived exertion (RPE) are commonly implemented to assess associated players internal response [19–22]. However, there is a lack of studies that have analysed the internal response to the peak and relative running demands of substitute players. Therefore, the aim of this study was to analyse the physical impact and internal response of substitute players, compared to starting and full-match players. This study also sought to determine if the differences between substitution statuses were influenced by playing position.

## MATERIALS AND METHODS

### Experimental approach to the problem

This study implemented a longitudinal observational study design. Data were collected on twenty-one (21) elite youth male soccer players during national (National Youth League, n = 8) and state (National Premier League, n = 21) competitive soccer matches (n = 29) across a 13-month period. National Premier League (NPL) competition matches are classified as semi-professional fixtures and were played throughout an eight-month competition calendar period (March – October). National Youth League (NYL) competition matches are classified as youth professional development fixtures and were played across a three-month competition calendar period (November – January). The running demands for each player during match-play were captured using a global positioning system (GPS). A rate of perceived exertion (RPE) was collected within 30-minutes of a player completing their match involvement to assess subjective match intensity. Players were categorised according to their substitution status and playing position in each match.

### Participants

Twenty-one (n = 21) outfield elite youth male soccer players (age: 15.6 ± 0.7 yrs.; height 173.1 ± 5.2 cm; body mass 64.9 ± 6.2 kg; Yo-Yo Intermittent Running Test (Level 2) score: 771 ± 220 m) from an Australian Under-17 National Centre of Excellence squad were monitored during 29 competition matches. A typical training week consisted of 4–5 soccer specific on-field sessions, 1–2 gym-based strength and conditioning sessions, 1–2 competition matches per week and 1–2 post-match contrast water immersion recovery sessions. All players and parents or guardians were informed of the risks and benefits of participation in this study prior to providing informed written consent. This study was approved by the Human Research Ethics Committee of the University of Canberra, Australia (Project Number: 16-195).

### Procedures

Each player competed in an average of 14 matches (range = 3–27), with a total of 300 individual match files used for analysis. Out of the 300 observations, 75 were for substitutes (players who replaced the starting players at some stage of the match), 76 were for starters (players who started the match and were replaced by a substitute player) and 149 were for full-match players (players who played the full 90-minutes of the match). Players were also categorised according to their position in each match and were categorised as: Central Attackers (CA, n = 33 observations), External Defenders (ED, n = 83 observations), Midfielders (MD, n = 105 observations) and External Attackers (EA, n = 79 observations). To account for participants featuring in different playing positions and substitution statuses across different matches, these were defined for each individual match accordingly. Central Defenders were not substituted during the data collection period and were therefore omitted from the analysis (n = 58 observations). Furthermore, players who were substituted in the last 5-minutes of match-play were omitted to minimise the effect of tactical substitutions made to delay match-play (n = 3 observations).

A 4-3-3 formation was used in all observed matches throughout the data collection period. Matches were preceded by a 30-minute standardised warm-up consisting of small-sided games, short and intermediate length maximal sprint efforts, short and long passing, shooting, and dynamic stretching. The field dimensions for matches were 100 × 60 m. Each match was 90-minutes in duration, separated into two 45-minute halves with any additional time determined by the referee. All matches were played under the same competition rules, limiting each team to three substitutions and a 15-minute break for half time. Competition match times and environmental conditions varied throughout the data collection period in accordance with NPL and NYL seasonal fixtures.

### Data collection

The physical demands of players during matches were captured using commercial 15 Hz GPS tracking devices (SPI HPU, GPSports, Canberra, Australia) according to manufacturer instructions. To minimise the effect of inter-unit error, each player was allocated the same GPS device for the duration of the data collection period. The inter-unit reliability for the GPS devices (expressed as a coefficient of variation) has been reported as 1.4% for total distance, 7.8% for distance at speeds between 2.0 m/s to 5.9 m/s, and 4.8% for distance covered at speeds > 5.9 m/s [23]. Each device was turned on 30-minutes before each match to ensure satellite connectivity, and between 4 to 12 satellites were available for connectivity and signal transmission during match-play, satisfying the criteria for ideal positional detection [24]. Horizontal dilution of precision (HDOP) was not reported by the proprietary software (Team AMS, Canberra, Australia). Raw GPS data files were extracted using proprietary software (Team AMS, Canberra, Australia) after the completion of each match. Data was trimmed so that only on-field playing time was included in the analysis.

Recorded GPS data included total distance covered (TD), and high-speed running distance (HSRD), defined as distance covered at speeds > 5.0 m/s. This threshold was similar to those used in previous investigations [7, 11]. To calculate relative running outputs, the absolute values of TD and HSRD were divided by the duration (in minutes) of the match the player was involved in. To calculate peak TD and HSRD, 1-, 2- and 5-minute rolling epochs were employed as fixed epochs have been demonstrated to underestimated total (7–10%) and high-speed (12–25%) distance (defined as > 5.5 m/s) in elite senior male soccer players [14]. Each match file was split into 30-second time intervals and 1-, 2-, and 5-minute rolling sums were then calculated. The peak TD and HSRD were then defined as the maximum TD and HSRD achieved in a 1-, 2-and 5-minute epoch. An example of this process is shown in Table 1. To allow for comparison between the different epoch lengths, peak TD and HSRD for 2- and 5-minute rolling epochs were expressed as relative per minute values, calculated by dividing the maximum distance achieved by the duration of the 2- and 5-minute epoch.

**TABLE 1:** Example of 1-, 2- and 5-minute rolling epoch calculations

30 second Time Splits	Distance	1-minute	2-minute	5-minute
0–30 sec	44			
30–60 sec	81	125		
60–90 sec	62	143		
90–120 sec	103	165	290	
120–150 sec	56	159	302	
150–180 sec	77	133	298	
180–210 sec	70	147	306	
210–240 sec	67	137	270	
240–270 sec	54	121	268	
270–300 sec	60	114	251	674
300–330 sec	57	117	238	687
330–360 sec	49	106	220	655
360–390 sec	88	137	254	681
390–420 sec	61	149	255	639
420–450 sec	64	125	262	647
450–480 sec	107	171	320	677
480–510 sec	91	198*	323*	698
510–540 sec	58	149	320	689
540–570 sec	64	122	320	699
570–600 sec	91	155	304	730*

* Denotes maximum distance achieved in 1-, 2- or 5-minute rolling epoch. Peak distance is calculated as the maximum distance achieved in a 1-, 2- and 5-minute epoch. For example, the maximum distance achieved for a 1-minute rolling epoch is from the 450–510 sec window. The maximum distance achieved for a 2-minute rolling epoch is from the 390–510 sec window and the maximum distance achieved for a 5-minute rolling epoch is from the 300–600 sec window. To allow for comparison between the different epoch lengths for peak TD and HSRD, 2- and 5-minute rolling epochs were expressed in relative per minute values, calculated by dividing the maximum distance achieved by the duration of the 2- and 5-minute epoch.

A rate of perceived exertion (RPE) was collected using a CR-10 scale to assess subjective match intensity [19–22]. The RPE was collected within 30-minutes of match completion, or within 30-minutes of a player coming off the field if they were substituted. For example, if a player was substituted during the 70^th^ minute of a match, that player completed the RPE CR-10 scale within the next 30-minutes. This method has been previously implemented to quantify subjective match intensity and is a valid monitoring tool in elite youth soccer [19–21].

### Statistical analysis

Statistical analyses were conducted using R version 4.0.3 [25] and RStudio version 1.4.1103 [26]. A Linear Mixed Model (LMM) was developed using the *lmer* function from the *lme4* package [27] to determine the difference in peak TD and HSRD (dependent variables) between substitution status and positions (fixed factors) for each epoch length. In addition, separate LMM’s were conducted to determine the difference in relative TD and HSRD (dependent variables) between substitution status and positions (fixed factors). A LMM was also performed to analyse the difference in RPE (dependent variable) for substitution status and positions (fixed factors). For each LMM, the repeated measures for each player were treated as a random factor. A Type II Wald F test was conducted using the *Anova* function from the *car* package [28] to determine the significance (alpha level = 0.05) of any interaction or main effects between substitution status and playing position. The assumptions of homoscedasticity and linearity were confirmed upon visual inspection of plots of the fitted values against the residuals [29]. The assumption of normality was determined upon visual inspection of histograms and Q-Q plots of the residuals. Effects size statistics were calculated by Cohen’s *d,* using the least squares means and the pooled standard deviation of the random effects to account for the structure of the LMM [30]. The effect sizes were interpreted as trivial: |*d*| < 0.2, small: |*d*| = 0.2– 0.49, moderate: |*d*| = 0.5–0.79 and large: |*d*| ≥ 0.8 [31]. Least-squares means and 95% confidence intervals were calculated using the *ls_means* function from the *lmerTest* package [27].

## RESULTS

**FIG. 1:**
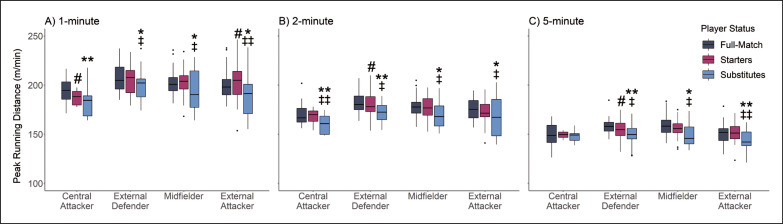
Peak Total Distance (TD) Characteristics According to Substitution Status and Position for (A) 1-, (B) 2- and (C) 5-minute rolling epoch lengths * |*d*| 0.2–0.49 small effect size between Substitutes to Full-match players. ** |*d*| 0.5–0.79 medium effect size between Substitutes to Full-match players. *** |*d*| ≥ 0.8 large effect size between Substitutes to Full-match players. # |*d*| 0.2–0.49 small effect size between Starting and Full-match players. ## |*d*| 0.5–0.79 medium effect size between Starting and Full-match players. ### |*d*| ≥ 0.8 large effect size between Starting and Full-match players. ‡ |*d*| 0.2–0.49 small effect size between Substitute and Starting Players. ‡‡ |*d*| 0.5–0.79 medium effect size between Substitute and Starting Players. ‡‡‡ |*d*| ≥ 0.8 large effect size between Substitute and Starting Players.

No interactions were identified between substitution status and playing position for peak TD across 1- (*p* = 0.863), 2- (*p* = 0.924) and 5-min (*p* = 0.645) rolling epochs and relative TD calculations (*p* = 0.283). Main effects were identified for substitution status for peak TD in 1- (*p* = 0.003), 2- (*p* = 0.004) and 5-min (*p* < 0.001) rolling epochs and relative TD calculations (*p* < 0.001). Figure 1 demonstrates that in 1- and 2-minute rolling epochs central attacker substitute players recorded less peak TD compared to full-match players. Similar results were found for external defenders and midfielders in 1-, 2- and 5-minute rolling epochs with substitute players reporting less peak TD compared to starting and full-match players. External attacker substitute players demonstrated lower peak TD compared to starting and full-match players in 1-, 2- and 5-minute rolling epochs.

Substitute players reported greater relative TD in comparison to full-match players for central attackers (Figure 3). Similar results were found for midfielders and external attackers with substitute players reporting greater relative TD running outputs in comparison to starting and full-match players (Figure 3).

**FIG. 2:**
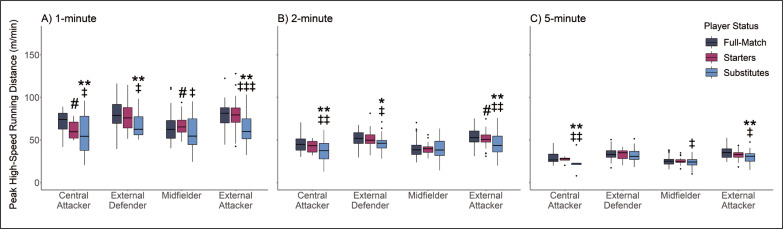
Relative High-Speed Running Distance (HSRD) Characteristics According to Substitution Status and Position for (A) 1-, (B) 2- and (C) 5-minute rolling epoch lengths * |*d*| 0.2–0.49 small effect size between Substitutes to Full-match players. ** |*d*| 0.5–0.79 medium effect size between Substitutes to Full-match players. *** |*d*| ≥ 0.8 large effect size between Substitutes to Full-match players. # |*d*| 0.2–0.49 small effect size between Starting and Full-match players. ## |*d*| 0.5–0.79 medium effect size between Starting and Full-match players. ### |*d*| ≥ 0.8 large effect size between Starting and Full-match players. ‡ |*d*| 0.2–0.49 small effect size between Substitute and Starting Players. ‡‡ |*d*| 0.5–0.79 medium effect size between Substitute and Starting Players. ‡‡‡ |*d*| ≥ 0.8 large effect size between Substitute and Starting Players.

**FIG. 3:**
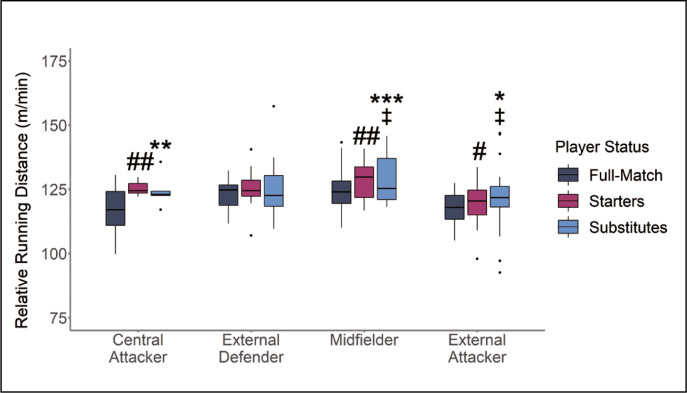
Relative Total Distance (TD) According to Substitution Status and Position * |*d*| 0.2–0.49 small effect size between Substitutes to Full-match players. ** |*d*| 0.5–0.79 medium effect size between Substitutes to Full-match players. *** |*d*| ≥ 0.8 large effect size between Substitutes to Full-match players. # |*d*| 0.2–0.49 small effect size between Starting and Full-match players. ## |*d*| 0.5–0.79 medium effect size between Starting and Full-match players. ### |*d*| ≥ 0.8 large effect size between Starting and Full-match players. ‡ |*d*| 0.2–0.49 small effect size between Substitute and Starting Players. ‡‡ |*d*| 0.5–0.79 medium effect size between Substitute and Starting Players. ‡‡‡ |*d*| ≥ 0.8 large effect size between Substitute and Starting Players.

No interactions were observed between substitution status and playing position for peak HSRD across 1- (*p* = 0.507), 2- (*p* = 0.591) and 5-min (*p* = 0.861) rolling epochs and relative HSRD calculations (*p* = 0.414). Main effects were also identified for substitution status for peak HSRD in 1- (*p* < 0.001) and 2- (*p* = 0.017) rolling epochs as well as relative HSRD calculations (*p* < 0.001). No main effects were identified for substitution status in 5-minute (*p* = 0.070) rolling epochs. Substitute players perform less peak HSRD in 1-minute rolling epochs compared to starting and full-match players in central attacker, external defender, and external attacker positions (Figure 2). Substitutes are reported to produce less peak HSRD compared to starting and full-match players in 2- and 5-minute rolling epochs for central attackers and external attackers.

**FIG. 4:**
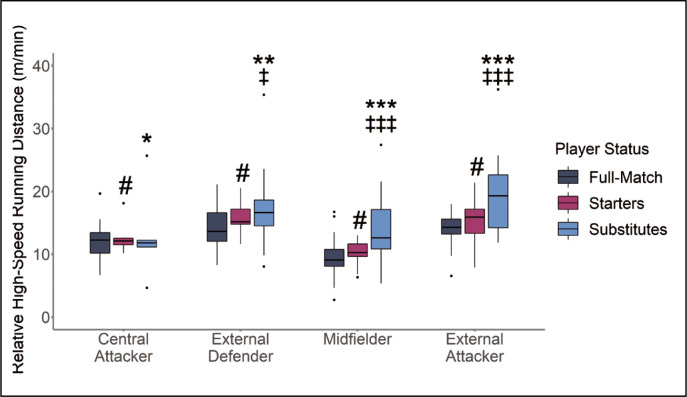
Relative High-Speed Running Distance (HSRD) According to Substitution Status and Position * |*d*| 0.2–0.49 small effect size between Substitutes to Full-match players. ** |*d*| 0.5–0.79 medium effect size between Substitutes to Full-match players. *** |*d*| ≥ 0.8 large effect size between Substitutes to Full-match players. # |*d*| 0.2–0.49 small effect size between Starting and Full-match players. ## |*d*| 0.5–0.79 medium effect size between Starting and Full-match players. ### |*d*| ≥ 0.8 large effect size between Starting and Full-match players. ‡ |*d*| 0.2–0.49 small effect size between Substitute and Starting Players. ‡‡ |*d*| 0.5–0.79 medium effect size between Substitute and Starting Players. ‡‡‡ |*d*| ≥ 0.8 large effect size between Substitute and Starting Players.

Within-positional differences for central attackers demonstrated that substitute players reported greater relative HSRD in comparison to full-match players (Figure 4). External defenders, midfielders and external attackers also displayed similar within-positional differences with substitute players reporting greater relative HSRD than starting and full-match players (Figure 4).

**FIG. 5:**
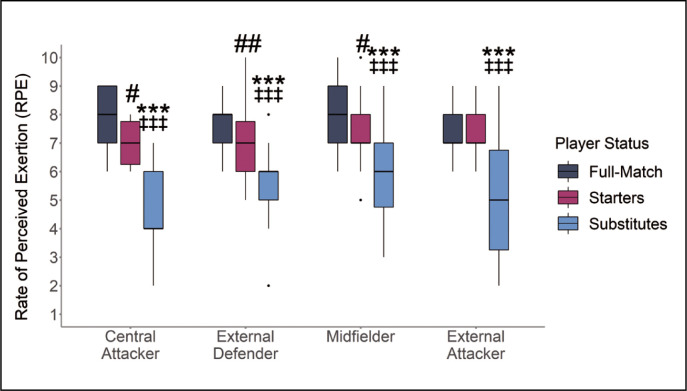
Reported Rate of Perceived Exertion (RPE) according to Substitution Status and Position * |*d*| 0.2–0.49 small effect size between Substitutes to Full-match players. ** |*d*| 0.5–0.79 medium effect size between Substitutes to Full-match players. *** |*d*| ≥ 0.8 large effect size between Substitutes to Full-match players. # |*d*| 0.2–0.49 small effect size between Starting and Full-match players. ## |*d*| 0.5–0.79 medium effect size between Starting and Full-match players. ### |*d*| ≥ 0.8 large effect size between Starting and Full-match players. ‡ |*d*| 0.2–0.49 small effect size between Substitute and Starting Players. ‡‡ |*d*| 0.5–0.79 medium effect size between Substitute and Starting Players. ‡‡‡ |*d*| ≥ 0.8 large effect size between Substitute and Starting Players.

No interactions were found between substitution status and playing position for RPE (*p* = 0.246). However, main effects were identified for substitution status (*p* < 0.001). Within-positional differences demonstrated that full-match players reported greater RPE scores than starting and substitute players for central attackers (Figure 5). Similar results were found for external defenders, midfielders and external attackers with full-match players reporting greater RPE scores than substitute players (Figure 5).

## DISCUSSION

This study investigated the physical impact and internal response of substitute players, compared to starting and full-match players. This study also determined if differences between substitution statuses were influenced by playing position. The results of this study demonstrate that, when analysed using peak metrics, substitute players generally produced lower peak total distance and peak high-speed running distance compared to starting and full-match players (Figures 1 and 2). In contrast, when analysed using relative metrics, substitute players across all playing positions recorded greater relative high-speed running distance compared to full-match players (Figure 4). Furthermore, the findings of this study report that starting and full-match players also recorded higher RPE compared to substitute players across all playing positions (Figure 5).

A substitute player’s ability to ‘come on and increase the speed of the game’ or ‘replace fatigued or underperforming players’ by superseding the physical demands of the player being replaced is perceived by coaches and practitioners to be a significant factor in determining the outcome of a soccer match [5, 6]. However, the results from the present study showed that substitute players did not supersede the peak running output of starting and full-match players. These results could be partly explained by pre-entry warm up strategies. For example, Hills et al. [18] observed that typical match-day substitute warm up protocols failed to maintain core body temperatures of substitute players prior to pitch entry and subsequently impacted physical performance at the time of pitch entry. However, half-time re-warm up interventions have been cited to maintain sprint performance in the first 15-minutes of match-play compared to typical match-day half-time periods [16]. Consequently, the reduced peak high-speed running demonstrated by substitutes could be attributed to lack of physical preparation prior to pitch entry to meet the peak demands of match-play. However, further research is warranted to determine if specific warm-up interventions adequately prepare substitute players to meet the most demanding scenarios of match-play. Specifically, the differences of intermittent warm-up activities through the first half, half-time and second half of match play versus timed pre-entry interventions. For example, it is common practice for coaches to have numerous planned substitute scenarios according to possible score-line situations, i.e., losing 2-1 with 20-minutes remaining or winning 3-0 with 35-minutes remaining. Such pre-planned substitutions may be more effectively prepared for pitch entry using targeted interventions 30-minutes prior to pitch entry as opposed to intermittent warm-up activities. Interventions to prepare substitute players for preconceived tactical scenarios should be considered for future investigations.

Despite recording less peak high-speed running, substitute central attackers, external defenders, midfielders and external attackers displayed greater relative high-speed running compared to their full-match counterparts (Figure 4). Substitutes are typically introduced in the second half, recording a 15% increase in relative high-intensity (> 4 m/s) running when introduced at half-time and a 25% increase when introduced at the 75^th^ minute of match-play compared to full-match players at the same time period [1,4,32]. Previous research has identified that player pacing behaviour may alter according to the knowledge of exercise duration [33]. For example, Ferraz et al. [33] demonstrated that short exercise durations elicit more aggressive pacing strategies in small-sided soccer games. As such, substitute players pacing strategies may differ to starting and full-match players in accordance with full knowledge of their density of time to perform and therefore reduced time to achieve an objective i.e., being substituted in the 75^th^ minute to win the game in the remaining time available [33]. Our results suggest that a substitutes knowledge pertaining to the density of time to make a physical impact promotes a more aggressive pacing behaviour, reflected in the increased relative high-speed running (Figure 4). Practically, this means that substitute players may employ aggressive pacing strategies to make a physical impact but fail to produce the same or greater peak running outputs as starting and full-match players [5, 6, 33]. A play-er’s ability to perform actions at high-speed are critical in defensive and offensive situations such as pressing an opposition player for the ball or beating a defender in goal scoring situations [34, 35]. Considering the majority of goals are scored in the last 15-minutes of match-play [36] and are preceded by high-intensity running actions [34, 35], the inability to replicate or supersede the peak high-speed running of starting and full-match players indicates that substitutes may not be prepared for the most demanding, critical match situations, and may fail to provide a physical impact and subsequently influence the outcome of a match.

Substitute players recorded lower perceived match-intensity (RPE) compared to starting and full-match players in all positions (Figure 5). Research by Haddad et al. [22] suggested that session duration partially influences session RPE scores, accounting for 3.2% variance, and time spent at high intensity (i.e., between 91 and 100% of HRmax) accounting for 22.1% of variance in RPE scores. The reduced RPE scores recorded by substitute players may therefore be reflective of the reduced peak high-speed running indicating that substitute players spend less time in high-intensity zones during match-play. However, the total match duration is likely the main contributor to how elite youth soccer players internally respond to external demands and report subjective exertion [37]. Differential ratings of perceived exertion such as local-muscular (sRPEmus) and central-respiratory (sRPEres) have been used to enhance understanding and contextualise how players respond to external loads in elite soccer [37]. For example, Los Arcos et al. [37] found that players who completed greater than 70-minutes of match-play, i.e., starting and full-match players, reported higher average sRPEmus of 7.4 compared to sRPEres of 6.4. Alternatively, players that completed less than 20-minutes of match-play, i.e., substitutes, reported sRPEmus scores of 3.3 and sRPEres scores of 4.4 [37]. The results of this study are in alignment with Los Arcos et al. [37] suggesting that starting and full-match players have a greater subjective perception of muscular strain when reporting RPE compared to substitute players who likely report RPE based on central respiratory exertion.

Practically, employing interventions to ensure players are adequately prepared for ‘substitute specific’ scenarios may aid substitute players in replicating or superseding the peak running demands of the player they replaced. In turn, this may ensure that substitute players are ready to perform in crucial situations and gain an advantage over the opposition. Furthermore, the data presented in this study does offers some evidence for coaches and practitioners when designing training benchmarks and post-match top-up conditioning sessions. For example, when implementing running drills to compensate for the lack of external load exposure post-game, the implementation of additional running drills with the intent to benchmark against the greatest peak running demands may aid in maintaining physical conditioning. This could be particularly important for players who are regularly omitted from the starting line ups, i.e., substitutes.

However, caution must be implemented when interpreting these findings and several limitations should be considered. Firstly, further investigation is required as the peak running output of substitute players is likely influenced by technical and tactical factors such as ball-possession metrics and opposition playing formations which are not accounted for in this study. Furthermore, future studies should seek to incorporate variables such as game status (i.e., win, draw, loss), score line at time of substitute entry, the number of substitutes utilised by each team as well as the location of the match (i.e., home versus away) and opposition team quality (i.e., team rankings). In addition, examining player training loads, RPE scores and wellbeing during the days preceding competition may further elucidate the subsequent impact of player fatigue and readiness on match running performance according to substitution status. Finally, regard should be given to the potential variance of results attributed to factors such as variation in match timing, environmental differences between matches and any extensive travel requirements.

## CONCLUSIONS

In conclusion, the main finding of this study is that substitute players perform more relative high-speed running, but less peak total and high-speed running, compared to starting and full-match players. The results of this study suggest that a substitutes knowledge pertaining to the reduced time to make a physical impact may cause substitutes to modulate their efforts, resulting in an increased pacing strategy compared to starting and full-match players. Despite this substitute players failed to replicate or supersede the peak running demands of starting and full-match players, which could partly be explained by pre-entry warm up strategies.

## Conflict of interest declaration

The authors declare no conflict of interest.
